# Mechanisms of endocrine resistance in hormone receptor-positive breast cancer

**DOI:** 10.3389/fonc.2024.1448687

**Published:** 2024-10-31

**Authors:** Yuan Gao, Yang Yu, Mingqing Zhang, Wenjun Yu, Lihua Kang

**Affiliations:** Cancer Center, The First Hospital of Jilin University, Changchun, Jilin, China

**Keywords:** breast cancer, hormone receptor-positive, endocrine treatment, resistance, mechanisms

## Abstract

Hormone receptor-positive breast cancer may recur or metastasize years or decades after its diagnosis. Furthermore, hormone receptor expression may persist in relapsed or metastatic cancer cells. Endocrine therapy is one of the most efficacious treatments for hormone receptor-positive breast cancers. Nevertheless, a considerable proportion of patients develop resistance to endocrine therapy. Previous studies have identified numerous mechanisms underlying drug resistance, such as epigenetic abnormalities in the estrogen receptor (ER) genome, activation of ER-independent ligands, and alterations in signaling pathways including PI3K/AKT/mTOR, Notch, NF-κB, FGFR, and IRE1-XBP1. This article reviews the mechanisms of endocrine resistance in hormone receptor-positive advanced breast cancer, drawing from previous studies, and discusses the latest research advancements and prospects.

## 1 Introduction

Statistics from the Global Cancer Research Institute indicate that in 2020, female breast cancer surpassed lung cancer as the most common malignant tumor, with approximately 2.3 million new cases diagnosed annually, and approximately 700,000 women dying from the disease each year (1). In 2013, the St. Gallen International Breast Cancer Conference released its pathological molecular classification, which can be divided into Luminal A, Luminal B, HER-2 overexpression, and basal-like subtypes. Luminal A is hormone-sensitive, effective for endocrine therapy, and has a better prognosis than the other types (2). Approximately 70% of breast cancers express estrogen receptors (3). Estrogen is a steroid hormone that binds to receptors besides its impact on the reproductive system. It also exerts effects on other aspects of its physiological role, including cardiovascular, water, and salt metabolism, as well as the central and motor systems (4). Traditional endocrine therapies include selective estrogen receptor (ER) modulators (SERMs), selective ER degraders (SERDs), and aromatase inhibitors (AIs). The treatment strategy involves a blockade of the biological functions of estrogen and estrogen receptors. This blockade provides a significant survival benefit for such patients. Endocrine therapy has demonstrated an effective rate of 40–80% in patients with hormone receptor-positive disease (5). Although approximately 30% of patients with early breast cancer respond to endocrine therapy, subsequent drug resistance is inevitable, and approximately 50% of patients with advanced breast cancer and metastasis do not respond to endocrine therapy (6). Endocrine therapy remains a significant challenge in patients who can overcome resistance. Primary endocrine resistance refers to recurrence and metastasis within 24 months of adjuvant endocrine therapy, or disease progression within 6 months of first-line endocrine therapy for metastatic breast cancer (7). Secondary endocrine resistance refers to other endocrine resistance conditions that do not conform to primary endocrine resistance (7). Endocrine drug resistance results from the interplay between multiple mechanisms. This review primarily focuses on the current nature or possible mechanisms and recent progress in drug resistance.

## 2 Estrogen receptor and ESR1

**Figure 1:**
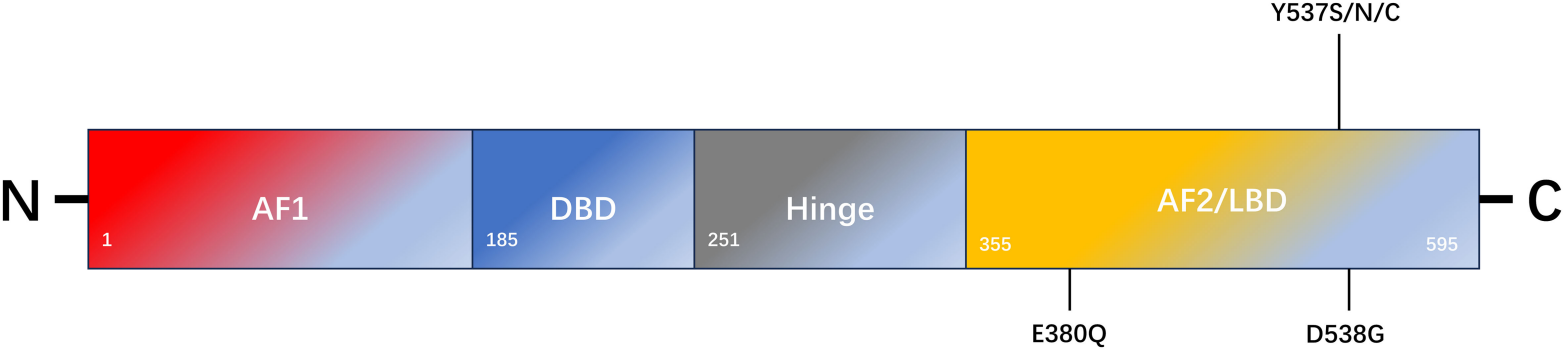
Schematic representation of ESR1 encoding ERα and the most common mutation sites of endocrine resistance. ERα comprises 595 amino acids. The structural domains of ERα include the transcription activation function 1 domain (AF1), DNA-binding domain (DBD), ligand-binding domain (LBD), AF2 domain, and flexible-hinge domain.

ERα and ERβ are expressed in numerous tissues, including the uterus, ovary, breast, prostate, lung, and brain (8). The DNA-binding domains (DBD) of ERα and ERβ exhibit 96% homology, whereas the ligand-binding domain (LBD) displays 53% sequence similarity (8). The primary distinction lies in their respective N-terminal hormone-independent transcriptional activation (AF-1) domains (8). In the context of breast cancer, ERα represents the primary manifestation and can be activated by 17-β-E2, which plays a pivotal role in regulating cell growth, proliferation, and migration, as well as other biological functions. ERβ is likely to be a protective factor that inhibits cell proliferation and plays an antitumor role (9). ERα is a 66 kDa ligand-dependent transcription factor composed of 595 amino acids, including one central DNA-binding region, one ligand-binding region, and two trans-active domains (10) (**Figure 1**). The A/B domain, at the amino terminus, encompasses the ligand-independent activation region AF-1, which is regulated by phosphorylation (11). The C domain is responsible for binding to estrogen response elements (ERE) and the DNA sequence of the target gene (11). The D-domain is a hinge region that contributes to the specificity and nuclear localization of DNA-binding (12). The E domain represents the ligand-dependent activation region of the LBD and the AF-2 region, and is regulated by estrogen or SERMs (11). The C-terminal helix H12 in the LBD is a pivotal component of AF-2 cleavage and determines the agonist or antagonist status of the receptor (13). The F domain was identified at the carboxyl-terminal end. The ER is activated to regulate the expression of numerous genes by directly binding to EREs within the nuclear genome or interacting with other transcription factors (11).

ERα and ERβ are located on different chromosomes and are encoded by ESR1 and ESR2, respectively. Mutations in these genes appear to be one of the main mechanisms underlying secondary endocrine resistance (14). In approximately 30% of metastatic hormone receptor-positive breast cancers, ESR1 mutations enhance the active conformational stability of ERα, particularly in patients who have been treated with AIs (15). An ESR1 mutation is an acquired mutation that results in ligand-independent ER activation (16). Studies have reported that mutations in the LBD region of ESR1 can be detected by next-generation sequencing in histological specimens of recurrent and metastatic hormone receptor-positive breast cancer (17, 18). The most common types of point mutations include the D538G (15%-20%), Y537S (5%-10%), and E380Q (5%-10%) mutations, which are in the LBD region of ERα (19, 20). Both the D538G and Y537S point mutations alter the H11-12 ring in the LBD region of ERα. The altered spatial conformation of H11-12 results in a structure that is more similar to that of the wild-type ERα-E2 complex, which maintains the receptor in an excited state. This pattern simulates the activated ligand-binding receptor pattern and blocks the binding of SERMs or SERDs to the receptor (21). These mutations result in structural changes at the protein level, which leads to a reduction in ligand affinity for the receptor-binding domain (21, 22). Keren Merenbakh-Lamin et al. conducted genetic analysis on tumor samples from 13 patients with metastatic breast cancer and examined the capacity of 538G-ERα to stimulate MCF-7 cell proliferation (23). The results demonstrated that compared to WT-ERα, 538G-ERα exhibited a 33% increase in cell proliferation in the untreated group and a 28% increase in the E2-treated group. Furthermore, 538G-ERα is more prone to distant metastasis (23). Previous studies have demonstrated that D538G and Y537S mutation models induced by doxycycline promote tumor metastasis. However, tumor cells in metastatic foci retreat after the inducer is withdrawn, which indirectly indicates that tumor metastasis is caused by mutations (24). Other studies have demonstrated elevated TCA activity in 537S-ERα mutants, which are not only glucose dependent but also use glutamine as an alternative carbon source compared to WT-ERα cells, which are primarily glucose dependent (25). Consequently, the mutants exhibited a heightened biological capacity to invade and metastasize, which may account for the prevalence of ESR1 mutations in metastatic breast cancer but not in early breast cancer. The Paloma-2 trial demonstrated that patients continued to accumulate the Y537S mutation throughout treatment with fulvestrant alone or combined with Palbociclib (26). In the Paloma-3 trial, patients with the Y537S mutation were treated with fulvestrant. The results demonstrated that the Y537S mutation had a worse clinical outcome than the D538G mutation (26). The combination of AI and mTOR inhibitors administered to patients with the D538G mutation has been shown to result in more favorable therapeutic outcomes than the Y537S mutation (20). In contrast, Jeselsohn et al. demonstrated that SERM or SERD treatment of the Y537S mutant exhibited a more pronounced anti-growth inhibition effect than that of the D538G mutant and wild-type ERα (24). This study revealed that cell lines treated with SERM or SERD for an extended period did not develop ESR1 mutations, whereas most mutations occurred during the withdrawal of AI drugs (24, 27). Spoerke et al. examined ESR1 mutants in 37% (57/153) of ctDNAs from patients before and after the progression of AI drug use using liquid biopsy (28). The researchers compared ctDNA with matched tumor tissue data and found that ESR1 mutations (0/81), (3/31), and (12/19) were present in tumor tissues collected at the initial diagnosis, before AI treatment, and after AI treatment progression, respectively. Furthermore, the content of ESR1 mutations in ctDNA is often higher than that in matched tumor tissues (28). Survival analysis revealed that the overall survival rate of individuals harboring the Y537S or D538G mutant was lower than individuals harboring the wild-type form of ERα (20.7 months vs. 32.1 months) (29). Nevertheless, preclinical studies indicate these mutations elicit disparate responses to SERMs and SERDs. For instance, they exhibit reduced sensitivity to fulvestrant, although this depends on dosage (30).

Transcriptional regulatory nucleoprotein 1 (NUPR1, P8, and COM-1) is a transcription co-regulatory factor induced by tamoxifen (TAM) in a time- and dose-dependent manner. Wang et al. demonstrated that NUPR1 can bind to ESR1 and regulate the transcription of BECN1, GREB1, RAB31, PGR, CYP1B1, and other genes involved in autophagy and drug resistance. Furthermore, they observed that the level of NUPR1 was significantly elevated in TAMR cells, and that its expression level was significantly correlated with postoperative survival time (31).

Gene rearrangement is a pivotal driver of a multitude of solid tumors (32). Similarly, in advanced hormone receptor-positive breast cancer, the fusion of non-coding promoter genes is a factor in relapse resistance (33). In 2018, Hartmaier et al. applied a novel algorithm to target sequencing genome structure rearrangement (RES) in three breast cancer cell lines and identified gene fusion transcripts using DNA pairing and RNA sequencing. The intra-frame fusion transcripts ESR1-DAB2 and ESR1-GYG1 were found in patients with supraclavicular lymph node metastasis and bone metastasis, respectively (34). Subsequently, the ctDNA of 9,542 breast cancer patients was subjected to further analysis, which revealed other concentrated gene fusion transcripts and transcripts with higher abundance in ER-positive metastatic breast cancer. Researchers postulated that at least 1% of MBC cases were associated with ESR1 gene fusion, with a 10-fold increase in ctDNA (34). To ascertain its function, further analysis of ESR1-DAB2, ESR1-GYG1, and ESR1-SOX9 fusion genes revealed that they all exhibited ligand-independent characteristics. Compared to the wild-type, ER with ESR1-DAB2 and ESR1-SOX9 fusion genes exhibited tenfold greater activity, whereas the ER activity of the mutant with LBD deletion was lower than the wild-type. However, these activities are independent of ligands (34). Elimination of the LBD region by the ESR1 fusion protein, which carries multiple 3’ chaperone genes, may be the mechanism underlying endocrine relapse drug resistance. Furthermore, drug therapies for ER are insensitive.

## 3 Cell cycle pathway: CDK4/6

**Figure 2:**
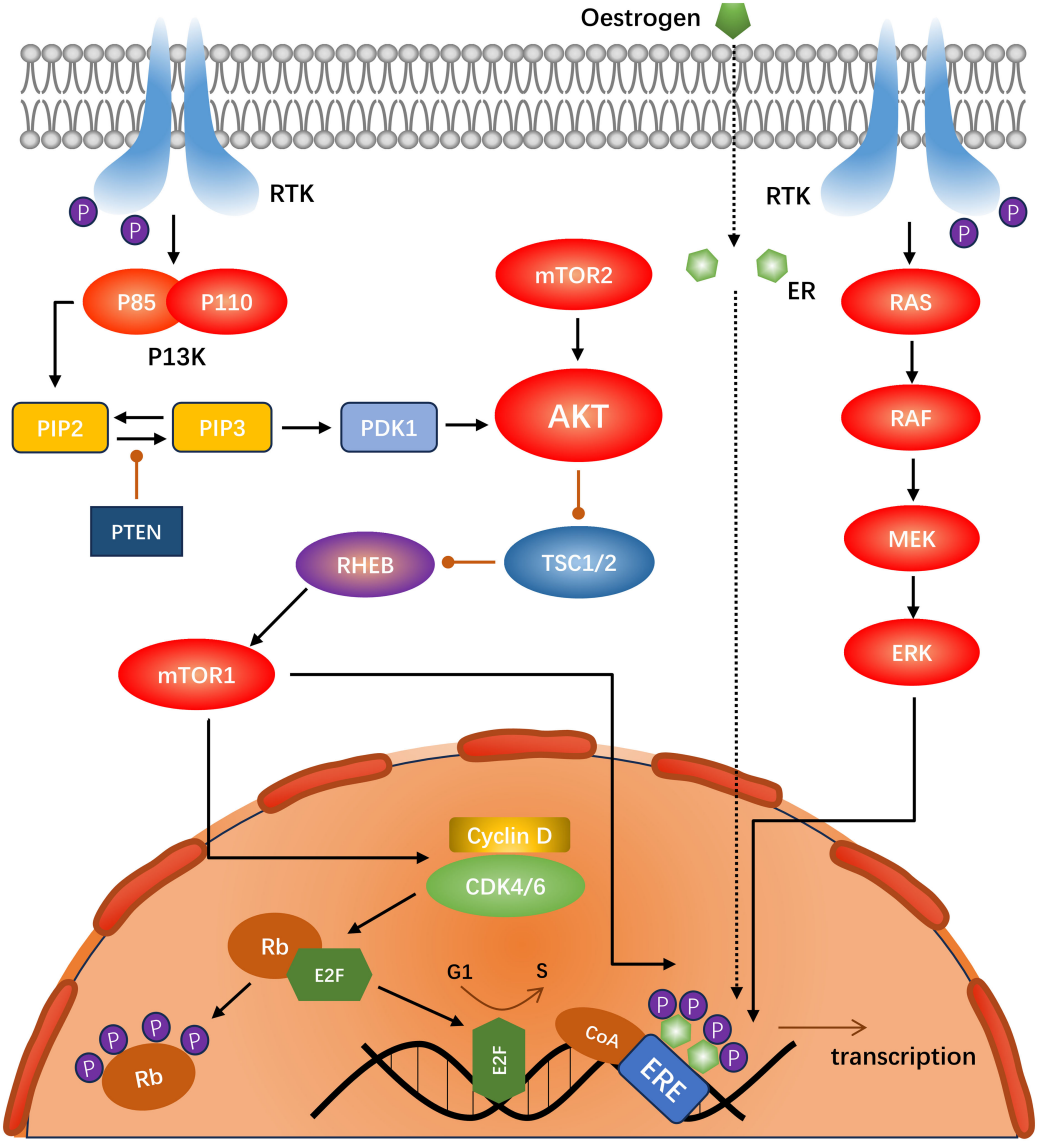
Schematic of the interactions between the PI3K-Akt-mTOR, RAS-RAF-MEK-ERK, and CDK4/6 pathways in ER-positive, HER2-negative breast cancer.

Cyclin-dependent kinase 4/6 (CDK4/6) has been identified as a key driver of ER-positive breast cancer growth and proliferation (35). Amplification and overexpression of cyclin D1 (encoded by CCND) are common in breast malignancies, with a particularly high prevalence in the Luminal A (29%) and Luminal B (58%) subtypes. In contrast, CDK4 is amplified in Luminal A (14%) and Luminal B (58%) subtypes (36). CyclinD1 binds to CDK4/6 to form a holoenzyme complex. In this process, the KIP/CIP protein (encoded by CDKN1) is required to assist in the assembly of the complex, whereas KIP/CIP inhibits the CDK1/2 complex (37). The holoenzyme complex phosphorylates a subset of the retinoblastoma protein (Rb) family, including P107 (encoded by RBL1), P110 (encoded by RB1), and P130 (encoded by RB2) during the G1 phase. The CDK2-cyclin complex then phosphorylates Rb (38), releasing the E2F transcription factor and inducing cyclin E (encoded by CCNE) to form a complex with other CDK1-3, which induces a series of biological reactions. Concurrently, the cyclin-CDK4/6 complex can directly phosphorylate the transcription factor FOXM1 (39), facilitating the transition of cells from G1 to S phase (40, 41) (**Figure 2**).

The activity of CDK is regulated by endogenous inhibitors; however, it requires the involvement of fully functional Rb proteins rather than incomplete or disabled Rb proteins (42). CDK inhibitors belong to the CDK-interacting protein/kinase inhibitor (CIP/KIP) family, which exerts both activating and inhibitory effects and influences the activity of cyclin-CDK complexes (43). This family of proteins includes p21CIP1, p27KIP1, and p57KIP2 (43). They are inhibitors of CDK2, both *in vitro* and under conditions of cell growth arrest (44). They are essentially disordered proteins that fold sequentially into cyclin and CDK to form a complex (45). Mice lacking p21 or p27 are susceptible to tumor formation (46, 47). Some studies have demonstrated that low expression of P21 can facilitate the formation of the CDK4 complex, whereas high expression of P21 exerts an inhibitory effect (48). Guiley et al. demonstrated that p27 allosterically activates the CDK4-Cyclin complex through a remodeling kinase. The recombinant CDK4-cyclinD complex containing p27 activity is insensitive to inhibitors such as Palbociclib, whereas p21 exhibits relatively low activity (49). The INK4 family of proteins, including p16INK4A, p14ARF, p15INK4B, p18INK4C, and p19INK4D, specifically interacts with the catalytic domain of CDK4/6. This process is initiated by inhibition of the binding of the aforementioned proteins to cyclin D, which inhibits the kinase activity of CDK4/6 (43). This results in the release of E2F and subsequent cell cycle arrest in the G1 phase (50). In HR+ breast cancer, there is often concomitant inactivation of RB1, amplification of cyclinD1 gene, and inactivation of CDKN2. However, RB1 inactivation is rare (51). Loss of RB1 may be a mechanism of resistance to CDK4/6 inhibitors in tumors that have lost RB1 function. Furthermore, alterations in the overexpression of cyclin D or cyclin E are expected to reduce therapeutic responsiveness in tumors that retain functional Rb (52). The loss of RB1 function and high levels of CCNE1 expression resulted in a decrease in ESR1 and PRG expression levels and hormone-dependent reactivity, which demonstrates that RB1 status is related to the growth and proliferation of hormone-dependent tumor cells (53, 54). Cyclin D levels are regulated by multiple cellular signaling pathways and can also be involved in cell cycle regulation as independent kinases, such as interactions with hormone receptors and transcription factors (55). Studies have demonstrated that the expression level of cyclin D1 is elevated in breast cancer stromal cells, prompting fibroblasts to secrete pro-inflammatory factors and osteopontin, facilitating tumorigenesis (56). The inhibitory protein encoded by CDKN2 competitively binds to CDK4/6 and induces conformational changes. Inactivation of the inhibitory gene results in the indirect enhancement of CDK4/6 activity (41), increasing the sensitivity threshold of cancer cells to CDK4/6 inhibitors, which induces drug resistance (57). Several resistances mechanisms have been implicated, with hormone-dependent cell signaling being the most susceptible to CDK4/6 inhibitors (51). CDKN1 has both inhibitory and activating effects on CDK4/6 cells. Previous studies have indicated that loss of ERα expression and ESR1 gene mutations are frequently found in drug-resistant tissues treated with CDK4/6 inhibitors combined with anti-estrogen drugs (58). However, these studies also suggested that the sensitivity of tumors with endocrine resistance to CDK4/6 is not related to ESR1 status (59).

CDK4/6 inhibitors play a positive role in tumor immunity. Goel et al. identified a mechanism related to the involvement of CDK4/6 inhibitors in immune evasion, which enhances antitumor immunity (60). The expression of HLA in CCND1-amplified tumor cells was upregulated by CDK4/6 inhibitors, which also activated the expression of retroviral elements in tumor cells treated with Abemaciclib. This results in increased dsRNA levels, driving interferon-stimulating gene (ISG) production, increasing the production of type III IFN, upregulating IL-2 to inhibit Tregs, and decreasing the number of peripheral Tregs and Treg/CD8+ ratio (60, 61). These regulatory methods are independent of tumorigenesis and enhance the ability of antigen-presenting and tumor-killing CD8+ T cells. The combination of Abemaciclib with anti-PD-L1 demonstrated enhanced immune effects, including dendritic cell maturation, cytokine activation, Th1/2 pathway activation, antigen presentation, and cell enhancement (60, 62).

Some alterations in the targets of the P13K-AKT/RAS-ERK signaling pathway are related to the resistance to CDK4/6 inhibitors. Amplification and mutation of AKT1 and AKT3 in the AKT pathway represent one such pathway, whereas PIK3CA mutation does not constitute a mechanism of drug resistance. The combination of CDK4/6 and PI3K inhibitors resulted in the regression of breast cancer grafts with PIK3CA mutations, and the combination of PI3K, AKT, and mTORC1/2 inhibitors demonstrated increased efficacy in multiple preclinical models of breast cancer. Wander et al. performed whole-exome sequencing of 59 samples and identified genomic alterations associated with hormone receptor-positive breast cancer, including 27 (65.9%) of 41 CDK4/6 inhibitor-resistant biopsies. In 9% of 41 CDK4/6 inhibitor-resistant biopsies, at least one of the following eight changes was observed: activating mutations and/or amplification of AKT1, KRAS/HRAS/NRAS, FGFR2, and ERBB2; amplification of CCNE2 or AURKA; biallelic disruption of RB1; and loss of ER (63). These changes were still present in small amounts in the susceptible cohort but were less abundant than in the resistant cohort (63). These novel findings provide insights into the potential mechanisms underlying CDK4/6 resistance.

CDK4/6 inhibitors (including Palbociclib, Ribociclib, and Abemaciclib) can block the cell cycle by inhibiting downstream signaling. Both domestic and foreign guidelines to treat hormone receptor-positive breast cancer recommend a CDK4/6 inhibitor combined with endocrine therapy as the preferred initial treatment for patients with luminal-type breast cancer following the development of endocrine therapy. A summary of clinical studies evaluating CDK4/6 inhibitors in HR-positive/HER2-negative breast cancers is shown in **Table 1**.

**Table 1:** Summary of randomized phase II/III clinical trials evaluating CDK4/6 inhibitors in HR-positive/HER2-negative Advanced or Metastatic BC (36).

Trial Name	Phase	Population	Treatment Arms	Sample Size	Primary Outcome (Exp vs. Ctrl Arm) HR (95% CI)
MONALEESA-2	III	AI-sensitive postmenopausal women with HR-positive/HER2-negative advanced or metastatic BC; no previous systemic therapy for ABC	Ribociclib + Letrozole		PFS 25.3 vs. 16 months
			vs.	668	(HR 0.568; 95% CI
			Letrozole + Placebo		0.457–0.704)
MONALEESA-3	III	AI-sensitive/resistant postmenopausal women with HR-positive/HER2-negative advanced or metastatic BC; 0-1 line of ET for ABC	Ribociclib + Fulvestrant		PFS 20.5 vs. 12.8 months
			vs.	726	(HR 0.593; 95% CI
			Fulvestrant + Placebo		0.480–0.732)
MONALEESA-7	III	AI-sensitive peri/premenopausal women with HR-positive/HER2-negative advanced or metastatic BC; no previous ET and up to 1 line of CT for ABC	Ribociclib + TAM/NSAI		PFS 23.8 vs. 13.3 months
			vs.	672	(HR 0.553: 95% CI
			TAM or NSAI + Placebo		0.441–0.694)
MONARCH-2	III	AI-resistant pre/postmenopausal women with HR-positive/HER2-negative advanced BC that progressed after ET; no previous CT for ABC	Abemaciclib + Fulvestrant		PFS 16.4 vs. 9.3 months
			vs.	669	(HR 0.553; 95% CI
			Placebo + Fulvestrant		0.449–0.681)
MONARCH-3	III	AI-sensitive postmenopausal women with HR-positive/HER2-negative advanced or metastatic BC; no previous systemic therapy for ABC	Abemaciclib + NSAI		PFS 28.1 vs. 14.7 months
			vs.	493	(HR 0.540; CI
			Placebo + NSAI		0.418–0.698)
PALOMA-1/TRIO-18	II	AI-sensitive postmenopausal women with HR-positive/HER2-negative advanced or metastatic BC; no previous systemic therapy for ABC	Palbociclib + Letrozole		PFS 20.2 vs. 10.2 months
			vs.	165	(HR 0.488; 95% CI
			Letrozole		0.319–0.748)
PALOMA-2	III	AI-sensitive postmenopausal women with HR-positive/HER2-negative advanced or metastatic BC; no previous systemic therapy for ABC	Palbociclib + Letrozole		PFS 27.6 vs. 14.5 months
			vs.	666	(HR 0.563; 95% CI
			Letrozole		0.461–0.687)
PALOMA-3	III	AI-resistant pre/postmenopausal women with HR-positive/HER2-negative advanced or metastatic breast cancer that progressed after ET	Palbociclib + Fulvestrant		PFS 9.5 vs. 4.6 months (HR 0.46; 95% CI 0.36–0.59)
			vs.	521	
			Fulvestrant + Placebo		
PEARL	III	AI-resistant postmenopausal women with HR-positive, HER2-negative metastatic BC	Palbociclib + ET		PFS 7.5 vs. 10 months (HR 1.09; 95% CI 0.83-1.44)
			vs.	601	
			Capecitabine		
MAINTAIN	II	Pre/postmenopausal women or men with HR-positive/HER2-negative advanced or metastatic BC who have progressed on an AI plus a CDK4/6 inhibitor (either Palbociclib or Ribociclib)	Palbociclib or Ribociclib) vs. Fulvestrant + Placebo	119	PFS 5.29 vs. 2.76 months (HR 0.57; 95% CI 0.39–0.85)
	
	
	
	
PACE	II	Pre/postmenopausal women or men with HR-positive/HER2-negative advanced or metastatic BC who have progressed on an ET plus a CDK4/6 inhibitor and up to 1 line of CT for ABC	Fulvestrant vs.	220	PFS 4.8 vs. 4.6 (HR 1.11; 95% CI 0.79-1.55) vs. 8.1 months (HR 0.75; 95% CI 0.50-1.12)
			Palbociclib + Fulvestrant		
			vs.		
			Palbociclib + Fulvestrant +		
			Avelumab		

Exp, experimental; Ctrl, control; HR, hazard ratio; CI, confidence interval; AI, aromatase inhibitor; HR, hormone receptor; HER2, human epidermal growth factor 2; BC, breast cancer; ABC, advanced breast cancer; PFS, progression-free survival; ET, endocrine therapy; CT, chemotherapy; TAM, tamoxifen; NSAI, nonsteroidal aromatase inhibitors.

According to the findings of the PALOMA-1 clinical study, the Food and Drug Administration (FDA) approved Palbociclib in February 2015 (57). Subsequently, in a Phase III clinical trial of a CDK4/6 inhibitor combined with an aromatase inhibitor, Palbociclib, Ribociclib, and Abemaciclib demonstrated superior clinical efficacy in progression-free survival (PFS), establishing the first-line treatment status of AI combined with CDK4/6. The Phase III clinical PALOMA-2 study demonstrated that the PFS in patients treated with Palbociclib plus letrozole was significantly longer than in those treated with letrozole alone (24.8 months vs. 14.5 months; hazard ratio (HR) = 0.56; *P <* 0.001). Furthermore, the objective response rate (ORR) was higher in the combination therapy group (55.3% vs. 44.4%) (64, 65). The Phase III MONALEESA-2 evaluation of first-line Ribociclib + letrozole demonstrated that the median PFS in the Ribociclib + letrozole group was significantly longer than that in the placebo group (25.3 months vs. 16.0 months; HR = 0.57; *P <* 0.001), indicating an improvement (66). The MONARCH 3 clinical study demonstrated that the combination of Abemaciclib and AI significantly prolonged PFS in patients with advanced breast cancer who had not previously received systemic therapy (28.2 months vs. 14.8 months; HR = 0.54; *P <* 0.001) and ORR (61% vs. 46%) (67).

## 4 Cell signaling pathways

### 4.1 PI3K-AKT-mTOR

The PI3K-AKT-mTOR pathway plays a pivotal role in the regulation of numerous physiological processes. It is also one of the most prevalent signal transduction pathways in malignant tumors, as evidenced by numerous studies (68). PI3K (encoded by PIK3CA), a dimer composed of the regulatory subunit P85 and catalytic subunit P110, is activated by the receptor tyrosine kinases (RTKs) and GPCRs (69, 70), which phosphorylate PIP2 to PIP3. This process is inhibited by the negative regulator PTEN, resulting in an increased intracellular PIP3 concentration (71). This prompts PDK1 to phosphorylate threonine on AKT (depending on mTORC2) (71). Activation of AKT inhibits dimerization of Tuberous Sclerosis Complex 1/2 (TSC1/2), a negative regulator of mTORC1. Consequently, AKT activates mTORC1 indirectly (70). The principal nodes in the P13K-AKT-mTOR signaling pathway are shown in **Figure 2**.

PIK3CA mutations are present in 20% to 50% of patients with breast cancer, including 35% of hormone receptor-positive patients. In contrast, AKT and PTEN mutations are more common in hormone-receptor-positive patients (72). PIK3CA mutations are primarily observed in specific regions within exons 9 and 20, which encode PI3K helix and kinase domains, respectively (73). The mutation of exon 9 enables P110 α to circumvent the inhibitory function of P85 through the SH2 domain; however, the mechanism of exon 20 remains unclear (74). Loss of PTEN protein is more prevalent than loss of PTEN mutations in patients with breast cancer (75, 76). Stemke-Hale et al. demonstrated that the AKT1-E17K mutation is restricted to breast cancers expressing both ER and PR, and confirmed that AKT1-E17K, PIK3CA, and PTEN mutations are mutually exclusive in breast cancer cell lines, similarly to other tumor types (72). However, PTEN loss and PIK3CA mutations were not mutually exclusive, which is consistent with previous results (77). PIK3CA mutation is associated with high expression of AKT1 and cyclinD1, whereas PIK3CA, AKT mutation, and PTEN loss seem associated with a favorable clinical prognosis (72, 77). In wild-type PIK3CA, the loss of PTEN protein was associated with increased AKT activation, whereas PIK3CA mutation was not significantly associated with the phosphorylation of PTEN protein or its downstream substrate (72). However, PIK3CA mutations were not significantly associated with prognosis in patients with ERα-positive breast cancer treated with tamoxifen (72, 77). Furthermore, Wander et al. observed alterations in PIK3CA in both sensitive and drug-resistant hormone receptor-positive breast cancer biopsies, suggesting that PIK3CA is unlikely to cause drug resistance (63). Despite the absence of evidence that PTEN loss causes PI3K activation, Stommel et al. found that RTK inhibitors can downregulate AKT, suggesting that AKT activation may be associated with the absence of PTEN (78). In a previous retrospective study conducted by Tokunaga et al., endocrine therapy was significantly less effective in AKT-negative patients (*P <* 0.01) than in 12 AKT-positive patients (33.4%) (79), suggesting that AKT activation is associated with poor clinical outcomes. Both PI3K and mTOR belong to the PI3K-related kinase (PIKK) superfamily and share similar domains, which allows for the simultaneous targeting of these two kinases by some inhibitory drugs (70). Previous studies have also observed that the P13K-AKT-mTOR pathway is activated in MCF-7 cells with stable 537S-ER expression or transient 538G-ER expression and that phosphorylation of AKT, mTOR, and downstream S6K is enhanced (25). This is a downstream marker of mTOR activation and predicts lower survival in breast cancer patients with high expression of hormone receptor-positive breast cancer undergoing endocrine therapy (80). The combination of mTOR and AIs has been the standard of care for ER+ advanced breast cancer; however, this treatment has not demonstrated an improvement in survival in clinical trials (81). In addition, approximately 23% of breast cancers exhibit a loss of PTPN12 protein, which causes a loss of the ability to downregulate growth factor signal transduction and predicts poor prognosis (82).

The objective was to target mutated genes in the PI3K-AKT-mTOR signaling pathway, including the PI3K inhibitors alpelisib and inavolisib, and the AKT inhibitors ipatasertib and capivasertib. Although these drug studies have demonstrated a therapeutic effect on endocrine-resistant breast cancer, alpelisib is only suitable for patients with PIK3CA mutations and has not yet been approved in China. In contrast, capivasertib has not been approved for domestic or foreign use.

The BOLERO-2 and PrE 0102 studies demonstrated the clinical efficacy of a second-line combination regimen based on evolimus (83). A total of 724 patients were included in the international multicenter Phase III clinical study BOLERO-2, which opened a new treatment window for patients with endocrine-resistant breast cancer. The results demonstrated that the combination of everolimus and exemestane prolonged the median PFS in postmenopausal patients with late-stage HR-positive/HER2-negative breast cancer who relapsed or progressed after AI therapy; the HR = 0.45 (84). In BOLERO-2, the median PFS was longer in the everolimus plus exemestane group than in the exemestane alone group (7.4 months vs 2.0 months; HR = 0.52). This study provides a new strategy for postmenopausal patients with ER-positive/HER2-negative breast cancer (85). Based on the established efficacy of CDK4/6 inhibitors as second-line treatments, the TRINITI-1 study sought to evaluate the efficacy of a combination of exemestane, Ribociclib, and everolimus in patients with HR-positive/HER2-negative advanced breast cancer who progressed after CDK4/6 inhibitor treatment. The clinical benefit rate of the three-drug combination regimen at the end of 24 weeks was 41.1%, and the overall population median PFS was 5.7 months (86). The MIRACLE study included 199 domestic, multicenter patients with breast cancer. The results demonstrated that the ORR of the combined group treated with evolimus was 50.0% and 39.3%, respectively, compared to the ORR of the letrozole group. The median PFS was 19.4 months for the combined group and 12.9 months for the letrozole group, respectively (87). **Table 2** lists inhibitors designed to target the P13K-AKT-mTOR pathway (88).

**Table 2:** P13K-AKT-mTOR pathway potency (88).

Inhibitor	Drug		Target
Pan-class I PI3K inhibitors			
	Buparlisib	BKM120	Pan-PI3K
	Pictilisib	GDC-0941	Pan-PI3K
	Copanlisib	BAY 80-6946	Pan-PI3K
	SAR245408	XL147	Pan-PI3K
	PX-866		Pan-PI3K
Isoform-specific PI3K inhibitors			
	Taselisib	GDC-0032	p110α
	Alpelisib	BYL719	p110α
	MLN1117		p110α
	BAY 1082439		p110α/β
	CH5132799		PI3Kα/γ
	GSK2636771		p110β
	AZD8186		p110β
	SAR260301		p110β
	Idelalisib	CAL-101	p110δ
	IPI-145		p110δ
	AMG319		p110δ
Dual-specificity PI3K/mTOR inhibitors			
	BEZ235		PI3K/mTOR
	GDC-0980		PI3K/mTOR
	PF-05212384		PI3K/mTOR
	PF-04691502		PI3K/mTOR
	GSK-2126458		PI3K/mTOR
	SAR245409	XL765	PI3K/mTOR
mTOR inhibitors, rapalogs			
	Sirolimus	rapamycin	mTOR
	Nab-rapamycin		mTOR
	Temsirolimus		mTOR
	Everolimus		mTOR
	Ridaforolimus		mTOR
mTOR inhibitors, catalytic			
	OSI-027		mTOR
	AZD2014		mTOR
	MLN0128		mTOR
	PP242		mTOR
	ML-223		mTOR

### 4.2 Notch

The Notch pathway is highly conserved and activated by receptor-mediated activation through signal-sending and signal-receiving cells (89). In the Notch signaling pathway, the cells that initiate the signaling process, referred to as “signaling-sending cells,” express five ligands for the Notch receptor, whereas the cells that receive the signal, or “signaling-receiving cells,” express four receptors for the Notch ligand in proximity to each other. The classic Notch signaling pathway is intimately associated with a multitude of biological functions in cancer cells (90). Upon ligand-mediated activation of the Notch receptor by a signaling cell, the extracellular domain of the Notch receptor (NotchEC) is endocytosed into the signaling cell, accompanied by the Notch ligand. The transmembrane domain (NotCHIC-TM) of the extracellular domain of the signaling recipient cell is cleaved by ADAM twice. Subsequently, the Notch extracellular domain is cleaved by the gamma secretase complex (GIS) to generate NotchIC. Subsequently, it combines with the transcription activators CSL and MAML1 to form the NotCHIC-MamL-CSL complex, initiating the transcription of Notch signaling target genes. Previous studies by Hao et al. demonstrated that Notch 1 can promote the expression of ERα target genes, including VEGFA, CD44, cyclinD1, C-myC, and PS2, in an E2-deficient culture medium (91). Notch3 plays a pivotal role in regulating ERα expression. Xiao-Wei Dou et al. demonstrated that Notch3 enhances ERα expression by binding to CSL elements within ERα promoters in cell lines. Furthermore, they observed a reduction in ERα gene and protein levels in McF-7 and T47D cells following Notch3 silencing (92). Notch signaling also plays a pivotal role in cancer stem cells. In a separate study, Sansone et al. demonstrated that inhibition of the IL6R/IL6-Notch3 pathway could restore ERα expression and render CD133hiCSCs dependent on ERα instead of the IL6/Notch3-Jagged1 pathway (93). Notch4 is inhibited when ERα activates target genes through classical e2-dependent pathways. Consequently, the Notch signaling pathway is activated when ER expression is downregulated or ER signaling pathway is inhibited (94). Furthermore, the Notch signaling pathway plays a pivotal role in tumor epithelial-mesenchymal transition (EMT). In an experiment conducted by Bui et al., high expression levels of mesenchymal marker proteins were observed in TAMR-MCF-7 cells, demonstrating that Notch4/STAT3 crosstalk plays an important role (95). Moreover, the Notch pathway is also associated with ESR1 mutations. Gelsomino et al. investigated the common ESR1 mutant types Y537S, Y537N, and D538G, and detected high expression of relevant molecules in the Notch signaling pathway in the mutants compared to the wild-type (96). These findings indicated that ESR1 mutations may contribute to drug resistance in cell lines by modulating the ER/Notch pathway.

### 4.3 NF-κB

Nuclear transcription factor kappa B (NF-κB) plays a pivotal role in endocrine drug resistance in breast cancer. Under normal conditions, NF-κB binds to its inhibitor IκBα to form homodimers or heterodimers. The classical activation pathway involves the action of inflammatory factors such as IL-1β and TNF-α, which initially activate TGF-β-activated kinase 1 (TAK1). This activates the IKK complex, which comprises IKKα (β) and NEMO. Subsequently, the serine residues of IκBα are phosphorylated, resulting in proteasome cleavage. The released NF-κB is then transferred from the cytoplasm to the nucleus and binds to its target genes, inducing transcription (97, 98).

Biswas et al. identified low NF-κB expression in HR+ breast cancer and subsequently demonstrated that the ER-dependent pathway inhibits NF-κB gene activation (99). This may indicate a comparable inverse correlation between ER and NF-κB expression in breast cancer cells, a relationship that has been well documented in the literature (100). Ruchi Nehra et al. observed that the expression level of P65 was elevated in ER+ cell lines exhibiting resistance to TAM endocrine therapy, accompanied by an augmented transcriptional activity of NF-κB and AP-1 (97). Following the administration of an NF-κB inhibitor, the transcription of NF-κB was found to decrease, as was the proliferation of drug-resistant cell lines (97). Kubo et al. compared ER+ breast cancer patients before and after endocrine therapy with AI, and observed increased NF-κB expression and induced resistance to endocrine drugs in breast cancer cells with disease progression after treatment (101). In conclusion, these results demonstrate increased NF-κB expression in breast cancer cells exhibiting ER-positive recurrence and/or endocrine resistance. As previously discussed, NF-κB can influence the sensitivity of breast cancer cells to endocrine drugs by regulating ERα expression.

The Zeste Homolog 2 Enhancer (EZH2) can be activated by certain inflammatory factors within the tumor microenvironment in a manner dependent on NF-κB, and the expression of ER was significantly increased following the silencing of EZH2 (102). As previously stated, NF-κB expression is negatively correlated with ER expression in breast cancer cells. Wang et al. previously demonstrated that RelB, a member of the NF-κB family, inhibits the expression of ER (103). The forkhead box O3a (FOXO3a) transcription factor binds to the ER promoter to initiate ER transcription. Phosphorylation of FOXO3a by filamentous threonine protein kinase C (PKC) results in inactivation of FOXO3a protein, which regulates the activity of the c-Rel transcription factor (104). It has been demonstrated that ER and p65 exist in protein complexes in DNA. Furthermore, inhibition of the NF-κB pathway can block cytokine-dependent p65 recruitment and enhance ER recruitment (98). Both E2 and NF-κB play a role in promoter regulation, and crosstalk between them affects the ability of ERα to activate its target genes (98). This demonstrates how NF-κB affects ER binding to DNA and, thus, ER activity. NF-κB regulates ERα transcriptional activity through both classical and non-classical pathways. The classical NF-κB pathway is activated in a cytokine-dependent manner, as previously described. Cytokines such as TNF-α can induce S118 phosphorylation of the ERK-dependent AF1 fragment of ER, which directly activates the ERE. This enhances the binding of ER to co-stimulators, including SRC3 and CBP/P300. Consequently, the ER becomes more sensitive to E2 and less sensitive to TAM (98). Conversely, IKK-α, produced by the non-classical pathway, can recruit the co-stimulator A1B1/SRC3 through the phosphorylation of S118 to form a transcription complex with ER, IKKα, and A1B1/SRC3, mediating the transcription of E2 (105). Following the binding of ER to TAM, a conformational change occurs, enabling ER to bind to nuclear receptor corepressor 1 (NCoR1). This results in the inhibition of histone deacetylation (106), which is associated with target genes. However, this process can be inhibited by IL-1β (107). However, its precise mechanism of action remains unclear. The interdependence of the ER and NF-κB pathways can rapidly downregulate miR-181a/b in microRNAs (miRNAs), helping to generate amplification loops and upregulation of target genes. This represents another crosstalk between the ER and NF-κB (108), which reveals the complexity between them.

### 4.4 FGFR

Fibroblast growth factor receptors (FGFRs) are members of the RTK superfamily (109, 110), which includes FGFR1-4. These receptors contain tyrosine kinase and transmembrane and extracellular domains. FGFR5 (FGFRL1), which lacks an intracellular kinase domain, binds to FGFs and prevents their interaction with other FGFRs (110). Fibroblast growth factors (FGFs) are secreted by tumor cells or stroma and can be classified into different types based on their homology, which is not the focus of this discussion. Heparin sulfate proteoglycans (HSPGs) stabilize the binding of FGF ligands to FGFRs, induce self-dimerization following receptor activation, phosphorylate the intracellular tyrosine kinase region, and activate downstream signaling pathways, such as PLC-IP3-DG-Ca2+, Ras-MAPK, PI3K-AKT, and JAK-STAT (111, 112). FGFR alterations include point mutations and gene fusion (113, 114). The most common of these is FGFR1 gene amplification (115), which causes endocrine resistance as ligand-dependent and ligand-independent pathways in approximately 15% of ER+ metastatic breast cancers (115–117). However, tumor cells are sensitive only to highly amplified FGFR1/2, and the underlying mechanism has been confirmed in multiple studies (118, 119). Luigi Formisano et al. observed that *in vitro* simulated ER+/FGFR1-amplified breast cancer cell lines were given AI drugs to simulate estrogen deprivation (LTED). This resulted in an increase in FGFR1 amplification as well as an increase in FGF3/4/19 and CCND expression. Furthermore, FOXA1 was found to promote FGFR1 nuclear expression through FGFR1 recruitment, driving estrogen-independent ERα transcription and inducing endocrine drug resistance (120). Servetto et al. investigated the relationship between FGFR1 and nuclear expression and demonstrated that FGFR1 nuclear expression induces non-estrogen-dependent cell growth, whereas cell lines exhibit reduced sensitivity to tamoxifen and fulvestrant (121). Potential mechanisms include the promotion of transcription of anti-estrogen resistance-related genes, binding to RNA polymerase II, and occupation of transcriptional promoter sites (121). Mao et al. demonstrated that FGFR1 and FGFR2 overexpression in the presence of FGF2 activated the MAPK and PI3K/AKT pathways, leading to fulvestrant and CDK4/6i resistance. However, this process can be reversed (122). Formisano et al. conducted a study analyzed the mechanism of FGFR1 resistance to CDK4/6 inhibitors. Their findings indicated that cyclinD1-mediated FGFR signal transduction plays a pivotal role in cell resistance to CDK4/6 inhibitors. Furthermore, they demonstrated that FGFR1 inhibition restored cell sensitivity to drugs (123). Although FGFR2 alteration is relatively uncommon in breast cancer, it is involved in endocrine resistance of tumor cells. It has been demonstrated that the FGF7/FGFR2 pathway enhances PI3K/AKT-mediated phosphorylation of ERα, enhancing drug resistance to SERM (124). Moreover, FGFR2 overactivation results in cross-resistance between the SERD and CDK inhibitors (122, 123).

### 4.5 IRE1-XBP1

The unfolded protein response (UPR) signaling pathway plays a pivotal role in maintaining the functionality of the ER. The UPR pathway increases the ER protein folding ability when there is damage to this ability; misfolded or unfolded proteins accumulate in the ER, stimulating the UPR-mediated activity of transcription factors. This reestablishes the ability of the ER to dispose of proteins. Alternatively, UPR may induce cytotoxic death (125, 126).

Where intracellular proteins must be produced in large quantities, the UPR signaling pathways are activated. For example, the estrogen pathway can induce the expression of target genes in breast cancer cells, resulting in the production of proteins that promote growth and proliferation (127). The activation of the stress sensor molecules IRE1, PERK, and ATF6, which are on the ER membrane, initiates activation of the UPR signaling pathway (128). Normally, the ER chaperone protein, GRP78, interacts with three sensors to inhibit its activity. In response to ENR stress, sensor molecules dissociate from chaperone proteins and are activated, initiating a signaling cascade that enhances the activity of relevant transcription factors (129). Activation of IRE1 results in mRNA-specific splicing of XBP1 to form XBP1-SMRNA, but not XBP1-UMRNA (130). Nevertheless, there is crosstalk between the IRE1-XBP1 pathway and estrogen, which can induce endocrine resistance in cancer cells. Inhibiting the UPR signaling pathway may help to reverse drug resistance. First, estrogen activates UPR signaling through the PLC-PIP2-IP3 pathway in an ENR stress-independent manner (128). ER can lead to the simultaneous upregulation of XBP1 and IRE1, which jointly promotes the production of XBP1-S (131). This results in the formation of a positive feedback loop between XBP1 and the ER. Concurrently, ER can form a complex with XBP1-S to enhance ligand-independent transcriptional activity (132). Both XBP1-S and XBP1-U have been shown to promote endocrine resistance in ER+ breast cancers. Increased expression of XBP1-SMRNA and protein was observed in endocrine-resistant breast cancer cells, which promoted SERM and SERD resistance (131). The current understanding of the mechanisms of drug resistance is as follows: (1) XBP1-S enhances the transcriptional activation of ER and NF-κB, promoting endocrine resistance through the NF-κB signaling pathway (133). (2) XBP1-S induces the production of NCOA3, whereas phosphorylated NCOA3 stimulates the expression of NF-κB and promotes endocrine resistance (134, 135). (3) XBP1-U can promote the degradation of transcription factors P53 and FOXO1 and enhance the activities of transcription factors NF-κB and ER (132, 133). (4) The ectopic expression of XBP1-S has been demonstrated to induce an increase in BCL2 protein expression levels and to promote the resistance of cells to endocrine stress (136).

## 5 Tumor microenvironment

In the early stages of tumor development, monocytes and macrophages are recruited into the tumor microenvironment. Tumor-associated macrophages (TAMs) are pivotal regulators of tumorigenesis and exhibit anti-inflammatory and other intricate regulatory functions that facilitate tumor growth in most cases (137). The abundance of these cells is closely related to several key processes, including tumor evasion, immune surveillance, neovascularization, invasion, metastasis, response to treatment, and poor prognosis (138–140). In human breast tumors, inflammatory mononuclear cells (IMCs) are recruited by binding chemokine (C-C motif) ligand 2 (CCL2), which is synthesized by tumor and stromal cells, to chemokine receptor 2 (CCR2), which promotes neovascularization and tumor cell infiltration (141). Tumor necrosis factor α (TNF-α) is an inflammatory mediator in the tumor microenvironment. It is primarily produced by the mononuclear macrophage system and plays a pivotal role in the inflammatory-tumor association mediated by the NF-κB pathway. In a co-culture of MCF-7 cells with macrophages, Castellaro et al. observed that although MCF-7 cells can induce TNF-α-treated macrophages (conditioned macrophages) to produce IL-6, IL-8, CCL5, TNF-α, and other inflammatory cytokines in the absence of E2 or in the presence of an ERα antagonist, endocrine resistance in breast cancer cells is promoted in a non-hormone-dependent manner via the TNF-α/IL-6 pathway (142). TNF-α induced a consistent increase in STAT3 expression in MCF-7 cells co-cultured with KG-1 compared to that in MCF-7 cells cultured alone. This effect was not inhibited (142). Simultaneous blocking of IL-6 and STAT3 resulted in a significant reduction in MCF-7 cell proliferation, suggesting that the IL-6/STAT3 pathway plays a key role (142).

Extracellular matrix (ECM), cancer-associated fibrocytes (CAFs) and cancer-associated adipocytes (CAAs) are all involved in the genesis and development of tumors (143, 144). CAFs in hormone receptor-positive breast cancer is closely related to drug resistance. In general, CAFs in breast cancer stroma stimulate tumor cell growth, promote angiogenesis, and induce immune regulation by producing multiple stimulatory factors (145). In hormone receptor-positive breast cancer, CAFs has been found to induce resistance to endocrine therapy by producing soluble factors, proteases, and β1 integrin (146). CD146 (MCAM) is a matrix surface marker (147). The study found that CD146-CAF inhibited ER expression in MCF-7 cells, reduced sensitivity to estrogen, and increased resistance to tamoxifen (148). However, the presence of CD146+CAF stimulated ER expression and maintained estrogen-dependent proliferation and sensitivity to tamoxifen (148). Bone morphogenetic proteins (BMPs) are essential for maintaining epithelial integrity and antagonizing epithelial to mesenchymal transition (149). GREMLIN1 (GREM1) is a secreted BMP antagonist that sequester BMP ligands and prevent their binding to receptors (150). Transforming growth factorβ(TGFβ) secreted by breast cancer cells, stimulated GREM1 expression in CAFs (151). GREM1 abrogated bone morphogenetic protein (BMP)/SMAD signaling in breast cancer cells, and also promoted the fibrogenic activation of CAFs (151). These processes enhance the invasive ability of cancer cells, so the treatment of GREM1 targets may improve the prognosis of breast cancer patients with high GREM1 expression. CAAs are also the main cellular components of the breast cancer microenvironment. Changes in the expression and secretion profile of inflammatory mediators in CAAs, such as increased secretion of chemokines CCL2, CCL5, IL-6, TNF-α, VEGF, leptin, etc, will further promote the proliferation and invasion of tumor cells and the formation of new blood vessels (144). In addition, through the dynamic interaction between breast cancer cells and CAAs, CAAs are induced to initiate the high tumor-promoting ability of metabolic reprogramming to support tumor cell proliferation, a process involving almost all nutrients (144). Furthermore, studies have found that exosomes transfer carcinogenic miRNAs (such as miRNA-144, miRNA-126 and miRNA-155) from breast cancer cells to fat cells in the tumor microenvironment, leading to their transformation into CAAs (152). These mechanisms become an important driver of disease progression, and targeting cancer-associated fat cells could lead to the development of potential drug-assisted anti-tumor therapies. In fact, for individuals, especially obese breast cancer patients, this goal can be more easily and effectively achieved through reasonable diet and appropriate exercise.

Programmed death receptor 1 (PD-1) is an immune checkpoint protein expressed on activated T cells, primarily in non-lymphocyte tissues and some immune cells in the surrounding environment of breast cancer. It mediates the inhibition of tumor-infiltrating lymphocytes and reduces the killing ability of T cells to tumor cells by binding to the ligand PD-L1 (153). Previous studies have demonstrated that the mRNA and protein expression levels of PD-L1 are significantly elevated in ERα-positive breast cancer cell lines and that ESR1 is negatively correlated with PD-L1 mRNA expression (154). Consequently, ERα may act as a negative regulatory factor influencing the expression level of PD-L1. Clinical studies have analyzed the efficacy of the PD-L1 antagonist pembrolizumab in ER+/Her2- advanced breast cancer patients, with an ORR of 12% (95% confidence interval (CI), 2.5–31.2%) and clinical benefit rate (CBR) of 20% (95% CI, 7–41%) (155).

It has been demonstrated that mesenchymal action on tumor cells can protect against cancer cell death (156). Pontiggia et al. discovered that soluble factors derived from fibroblasts, including matrix metalloproteinases (MMPs) and growth factors, are involved in the paracrine induction of drug resistance in tamoxifen-sensitive breast cancer epithelial cells through the PI3K/AKT and EGFR pathways. This was demonstrated by studying cultured fibroblast LM05-F and epithelial cell LM05-E (146). (2) Fibronectin and β1 integrin induce drug resistance in TAM by activating the downstream MAPK/ERK1/2 and PI3K/AKT pathways. (3) The phosphorylation of ERα-specific serine in epithelial cells by stroma-derived soluble factors and fibronectin is associated with tamoxifen (TAM) resistance in breast cancer. Furthermore, Sampayo et al. demonstrated that fibronectin mediates the endocytosis of ERα in breast cancer cells, with a subset of these cells entering the nucleus and the remainder being dragged back to the cell surface by β1 integrin. This evidence supports the critical role of the β1 integrin/FN pathway in regulating ERα expression (157). Heather M. Brechbuhl et al. demonstrated that CD146-CAFs inhibited ERα expression in MCF-7 cells, whereas CD146+CAFs not only induced ERα expression but also restored the sensitivity of epithelial cells to TAM in conditioned medium (148). Fibroblast stromal cells from the bone marrow (hs5-CM) have been shown to mediate endocrine resistance in breast cancer cells by downregulating ER levels via the paracrine signaling pathway (158).

## 6 Antibody-drug conjugation

**Figure 3:**
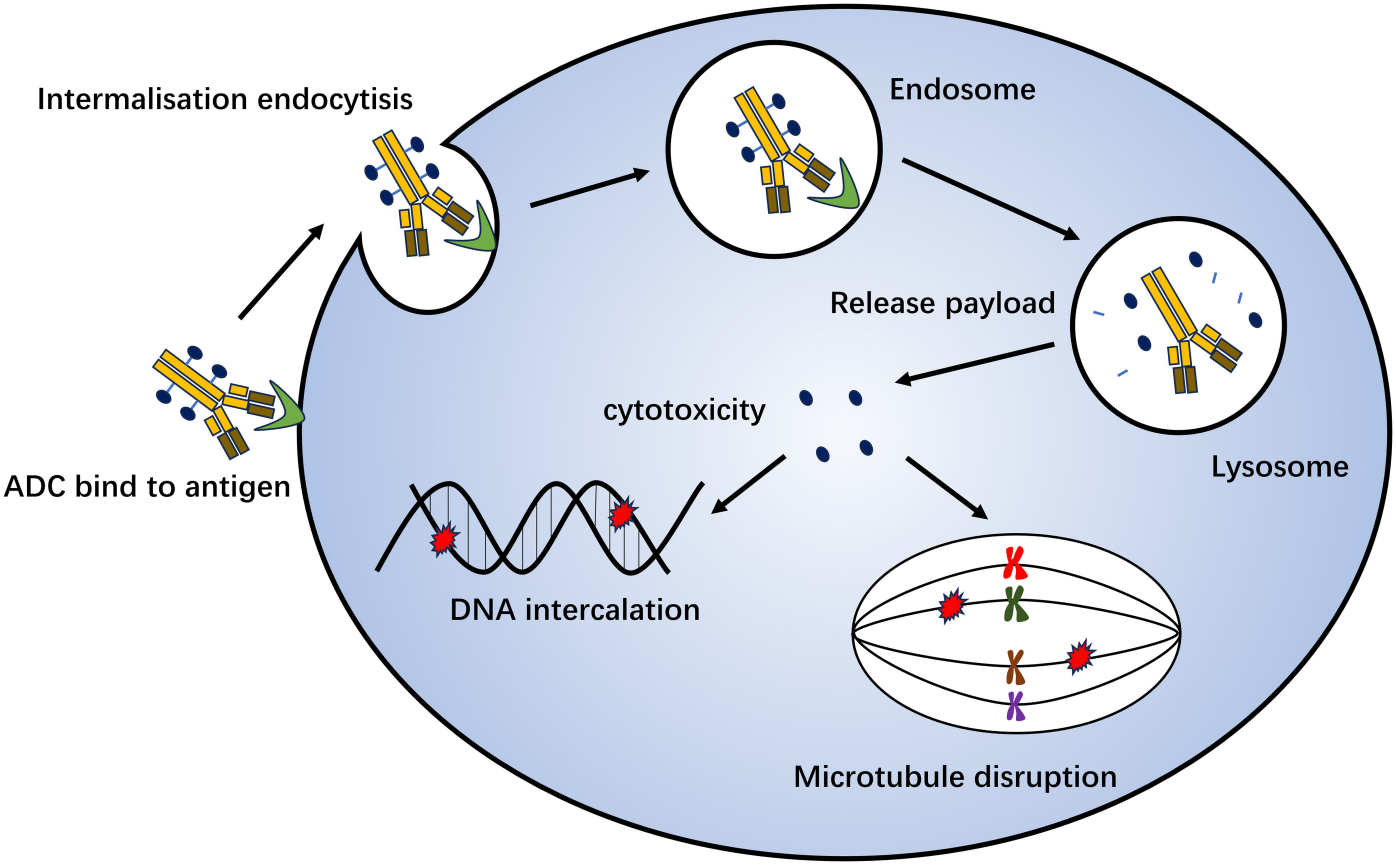
Mechanism of antibody-drug conjugation killing of tumor cells. Monoclonal antibodies on ADC can bind to specific target antigens on the tumor surface, enter tumor cells through receptor-mediated endocytosis, form early endosomes, and quickly release drugs. It can also mature into late-stage endosome and lysosome fusion and release drug loading, which ultimately leads to tumor cell death by inhibiting microscopic polymerization or DNA assembly.

Antibody-drug conjugation (ADC) is a novel antitumor drug that has emerged in recent years. It involves joining a monoclonal antibody with a drug carrier through a linker. Monoclonal antibodies on ADC can bind to specific target antigens on the tumor surface, enter tumor cells through receptor-mediated endocytosis, form early endosomes, and rapidly release drugs. Furthermore, they can mature into late-stage endosome and lysosome fusion and release drug loading, ultimately leading to tumor cell death by inhibiting microscopic polymerization or DNA assembly (159) (**Figure 3**). ADC drugs have made remarkable clinical progress in the field of breast cancer. Common research targets include the trophoblast cell surface antigen 2 (Trop-2), HER2, HER3, poliovirus receptor 4, and receptor tyrosine kinase-like orphan hormone receptor 2 (**Table 3**).

**Table 3:** Antibody-drug conjugation in HR-positive/HER2-negative Advanced or Metastatic BC.

Trial Name	Phase	Population	Treatment	Sample Size	Primary Outcome, HR (95% CI)
DESTINY-Breast04 (160)	III	HR+ disease considered endocrine refractory	T-DXd		PFS 10.1 vs. 5.4 months
		HER2-low(ICH 1+ vs 2+/ISH-), unresectable, and/or	vs.	557	(HR 0.51; 95% CI 0.40–0.64)
		mBC treated with 1-2 prior lines of chemotherapy	TPC		OS 23.9 vs. 17.5 months
		in the metastatic setting			(HR 0.64; 95% CI 0.48–0.86)
TROPiCS-02 (161, 162)	III	Metastatic or locally recurrent inoperable HR+/HER2-	SG		PFS 5.5 vs. 4.0 months
		breast cancer that progressed after	vs.	543	(HR 0.66; 95% CI 0.53–0.83)
		at least 1 endocrine therapy, taxane, and CDK4/6 inhibitor in any setting	TPC		OS 14.4 vs. 11.2 months
		at least 2, but no more than 4, lines of chemotherapy for metastatic disease			(HR 0.79; 95% CI 0.65–0.96)
TROPION-Breast01 (163)	III	Adult pts with inoperable or metastatic HR+/HER2-BC,	Dato-Dxd		PFS 6.9 vs. 4.9 months
		who had experienced progression on endocrine therapy	vs.	732	(HR 0.63; 95% CI 0.52–0.76)
		and for whom ET was unsuitable, and who had received	ICC		OS-
		1-2 prior lines of systemic chemotherapy			
SKB264 (MK-2870) (164)	II	pre-specified subpopulation of patients with HR+/HER2 mBC	SG	54	PFS 5.5 months (95%CI 3.6~7.6)
		from the phase 1/2, single-arm trial (NCT01631552)			OS 12.0 months (95%CI 9.0~18.2)

T-DXd-trastuzumab deruxtecan; SG-sacituzumab govitecan; TPC-treatment of physician’s choice; ICC-investigator’s choice of chemotherapy.

Trastuzumab emtansine (T-DM1) and trastuzumab deruxtecan (T-DXd) are ADCs that target HER2. Trastuzumab is the antibody component, and the drug antibody score is 3.5 for T-DM1 and 8 for T-DXd. In the Phase III clinical DESTINY-Breast04 study, 557 patients with HR-positive or HR-negative metastatic breast cancer with low HER2 expression who had previously received endocrine, first- or second-line chemotherapy were enrolled. Among patients with HR-positive disease, the T-DXd group was compared to the treatment of the physician’s choice (TPC) group. The chemotherapy regimens used in the TPC group included alibrine, capecitabine, albumin-paclitaxel, gemcitabine, and paclitaxel. The results demonstrated that the median overall survival (OS) of the T-DXd group and the TPC group were 23.9 months and 17.5 months, respectively (HR= 0.64). Furthermore, the median PFS was 10.1 months and 5.4 months, respectively (HR = 0.51). The efficacy of T-DXd is satisfactory and its overall safety profile is manageable (160).

Gosatuzumab (SG), Dato-DXd, and SKB264 are ADCs target Trop-2. The Phase III TROPiCS-02 study included 543 patients with HR-positive/HER2-negative metastatic breast cancer. SG demonstrated a significant improvement in median PFS compared to TPC (5.5 vs. 4.0 months; HR = 0.66; *P =* 0.0003), as well as an advantage in median OS (14.4 months vs. 11.2 months; HR = 0.79; *P =* 0.02) (161, 162). The Phase III TROPION-Breast01 study demonstrated that the Dato-DXd group exhibited superior PFS in previously treated HR-positive/HER2-negative metastatic breast cancer patients compared to the chemotherapy group (6.9 months vs. 4.9 months; *P <* 0.0001) (163). A Phase II study of SKB264 also demonstrated favorable antitumor effects, with a median follow-up period of 8.2 months, an ORR of 36.8%, and a median PFS of 11.1 months (164).

## 7 Biological metabolism

It has been demonstrated that abnormal endogenous lipid metabolism can cause increased cancer cell invasiveness and the development of drug resistance in tumors (165, 166). Fatty acid synthetase (FASN) is a key enzyme involved in lipid biosynthesis and the synthesis of long-chain fatty acids such as palmitate, which is subsequently involved in cell signal transduction (166, 167). FASN was initially identified as a highly expressed tumor marker in breast cancer (168). Studies have demonstrated that FASN plays an important role in the regulation of ERα expression and activity. Aleksandra Gruslova et al., building upon previous research, demonstrated that the inhibition of FASN in endocrine-resistant breast cancer cells induces endoplasmic reticulum stress (EnRs pathway), which mediates ERα degradation, resulting in a significant decrease in ERα levels in tumor cells (*P <* 0.01) and the inhibition of the growth of TAM-resistant breast cancer cells (169). In their experiments, Menendez et al. demonstrated that FASN controls the sensitivity of cells to E2-dependent ERα signals through the crosstalk of MAPK/ERα and AKT/ERα signals (165). Furthermore, it induces the expression of p21WAF1/CIP1, p27Kip1, and other cell cycle suppressor genes, inhibiting PI3K/AKT-mediated cell cycle progression and synergistically inhibiting E2-mediated cell survival (165). The etiology of breast cancer is multifactorial, with genetic susceptibility and environmental factors contributing to its pathogenesis.

## 8 Discussion

Treatment of hormone receptor-positive breast cancer has long been complicated by endocrine drug resistance. A considerable number of studies have identified numerous potential mechanisms and confirmed that the process of inducing drug resistance is complex. A multitude of studies on the molecular mechanisms have yielded new insights and novel therapeutic strategies that may overcome endocrine resistance.

Drugs are being developed to effectively block the transmission of estrogen signals and the activation of downstream molecules by regulating the expression and activation of ER and downstream signaling molecules. The previous treatment strategy was single estrogen antagonist therapy. Studies have demonstrated that Alterations in the ER genome play a pivotal role in the development of resistance to endocrine drugs. ESR mutants frequently exhibit drug resistance and distant metastases owing to their substantial aggressiveness. Single hormone receptor blockers have been ineffective in inhibiting tumor cell growth. In contrast, the combination of receptor blockade with downstream signaling molecule inhibitors has been shown to have antitumor effects. Combination therapy is often the recommended treatment for patients with hormone receptor-positive tumors. Endocrine resistance is not solely because of the “surface” molecular effect, but also encompasses the transmission of downstream signals. Of these, the RTK signaling pathway is of particular importance. Abnormal activation of this pathway leads to continuous activation of nuclear target genes and affects the expression level of ER. Consequently, activation of the RTK-mediated cell signaling pathway is largely associated with endocrine therapy resistance, which inhibits signal transduction and cell growth by inhibiting key targets. The intricate interrelationship between the ER signaling pathways and TKR, along with its downstream key PI3K-mTOR and RAS-ERK pathways, is a crucial aspect of endocrine resistance. In treatment-resistant endocrine-resistant breast cancer, ER can promote tumor growth and proliferation in a ligand-independent manner, replacing its classical activation pathway with a new mode influenced by other signal transduction pathways. Consequently, the development of drugs targeting downstream signaling molecules and a synergistic model combining them with endocrine therapy are anticipated to offer new hope for individuals with endocrine-resistant breast cancer.

The growth of tumor cells is also influenced by a multitude of factors, including intracellular and intracellular regulatory cytokines, immune molecules, tumor microenvironment, and stem cells. Further research into the immune microenvironment, oxidative stress space, and metabolome polymorphisms around tumor cells will help to elucidate novel mechanisms and linkages of drug resistance in tumors. Future research should aim to elucidate potential biomarkers in greater depth, identify more reliable targets, and develop more drugs for individuals resistant to frontline treatment. Individualized treatment was developed based on individual differences among the patients. A multitarget crossover model is expected to reverse endocrine resistance.

Previously, it was believed that breast cancer lacks immunogenicity. Over the years, immunotherapy has emerged as a new standard of care, demonstrating efficacy and therapeutic value in patients with tumors. CTLA-4, PD-1, and PD-L1 enhance the ability of immune cells to kill tumors by blocking immunoregulatory proteins that downregulate the immune system. Currently, medical evidence regarding the efficacy of immunotherapy for breast cancer is insufficient. Several tumor-infiltrating lymphocytes (TILs) and PD-L1 proteins may render TNBC sensitive to checkpoint inhibition (170). Nevertheless, the efficacy of immunotherapy in ER+ breast cancer remains unclear. Consequently, the combination of endocrine therapy and immunotherapy may represent a promising avenue for future research. Given the urgent need for further research into the role of immune checkpoints in endocrine resistance, it is imperative that clinical studies are conducted to determine the clinical benefits of combining endocrine and immunotherapies.

In recent years, macromolecular monoclonal antibodies, a novel class of targeted drugs represented by T-DXd, have emerged as a prominent area of research, ushering in the era of ADC drug therapy and offering expanded treatment options for advanced breast cancer patients who have progressed following CDK4/6 inhibitor treatment. The future of ADC drug research and development will continue to offer significant opportunities for improvement, including enhanced targeting, linker stability, and resistance to drug-induced toxicity.
